# High-Yield Production of Nano-Lateral Size Graphene Oxide by High-Power Ultrasonication

**DOI:** 10.3390/ma14081916

**Published:** 2021-04-12

**Authors:** Licínia Timochenco, Raquel Costa-Almeida, Diana Bogas, Filipa A. L. S. Silva, Joana Silva, André Pereira, Fernão D. Magalhães, Artur M. Pinto

**Affiliations:** 1 LEPABE, Faculdade de Engenharia, Universidade do Porto, 4200-180 Porto, Portugal; up201809122@fe.up.pt (L.T.); dianabogas@gmail.com (D.B.); fdmagalh@fe.up.pt (F.D.M.); 2 i3S—Instituto de Investigação e Inovação em Saúde, Universidade do Porto, 4200-180 Porto, Portugal; rcalmeida@i3s.up.pt (R.C.-A.); flsilva@i3s.up.pt (F.A.L.S.S.); 3 INEB—Instituto de Engenharia Biomédica, Universidade do Porto, Rua Alfredo Allen, 208, 4200-180 Porto, Portugal; 4 IFIMUP and IN-Institute of Nanoscience and Nanotechnology, Departamento de Física e Astronomia da Faculdade de Ciências da Universidade do Porto, 4169-007 Porto, Portugal; asilva.joana@gmail.com (J.S.); ampereira@fc.up.pt (A.P.); 5 CFP, Department of Physics Engineering, FEUP, Rua Dr. Roberto Frias, 4200-465 Porto, Portugal

**Keywords:** graphene, graphene oxide, particle size, stability, standardization, surface chemistry, nanomaterials

## Abstract

Nanographene oxide (GOn) constitutes a nanomaterial of high value in the biomedical field. However, large scale production of highly stable aqueous dispersions of GOn is yet to be achieved. In this work, we explored high-power ultrasonication as a method to reduce particle size of GO and characterized the impact of the process on the physicochemical properties of the material. GOn was obtained with lateral dimensions of 99 ± 43 nm and surface charge of −39.9 ± 2.2 mV. High-power ultrasonication enabled an improvement of stability features, particularly by resulting in a decrease of the average particle size, as well as zeta potential, in comparison to GO obtained by low-power exfoliation and centrifugation (287 ± 139 nm; −29.7 ± 1.2 mV). Remarkably, GOn aqueous dispersions were stable for up to 6 months of shelf-time, with a global process yield of 74%. This novel method enabled the production of large volumes of highly concentrated (7.5 mg mL^−1^) GOn aqueous dispersions. Chemical characterization of GOn allowed the identification of characteristic oxygen functional groups, supporting high-power ultrasonication as a fast, efficient, and productive process for reducing GO lateral size, while maintaining the material’s chemical features.

## 1. Introduction

Graphene oxide (GO) with very small lateral dimensions, in the order of 100 nm or less—commonly designated as nanographene (GOn)—has been attracting increasing attention in the biomedical field, particularly in the area of cancer treatment. Proposed applications include uses as drug carriers, platforms for photothermal and photodynamic therapies, or agents for biological imaging [1,2,3]. These take advantage of GOn’s physicochemical properties in combination with good biocompatibility and low toxicity. GO has been also explored to improve biomaterials’ physicochemical and biological properties [4] as well as for its antibacterial effect [5,6,7].

The size of the GO platelets is a relevant parameter when considering biological systems, since it may affect absorption into the body, penetration into blood vessels, cellular uptake, renal clearance, and selective toxicity [2,8,9]. GO is most widely produced using the modified Hummers method, a graphite chemical oxidation/exfoliation process, yielding sheets with lateral dimensions of tens or hundreds of microns. Decreasing the GO lateral dimensions to average values around 100 nm, ideally guaranteeing sufficiently narrow size distributions and maintaining the surface chemistry, in terms of degree of oxidation (i.e., avoiding reduction), has been the subject of several studies. These were recently reviewed by Tufano et al. [2] and the main approaches involved can be summarized as:

- Intensive oxidation of graphite by increasing concentration of oxidizing agents or increasing the timescale/cycles of oxidation process
- Centrifugation of GO in aqueous dispersion and separation of the fractions with smaller dimensions
- Breakdown of GO sheets by high-power ultrasonication
- Selective precipitation of larger GO sheets by protonation with organic solvents or by pH adjustment
- Exfoliation of graphite nanofibers with very small diameter
- Electrical breakdown of graphite by arc-discharge
- Ball milling of graphite in the presence of oxidizing agents
- Electrochemical exfoliation of graphite electrodes

Among these, electrochemical exfoliation has attracted attention for being relatively simple and fast [10]. However, ultrasonic treatment is also of particular interest since it has the potential to be a relatively expedited process that does not imply additional environmentally aggressive reactants or complex manipulation and can be easily scaled up. Ultrasonication is actually already used in combination with the Hummers or Marcano methods of GO production to achieve the final exfoliation and dispersion of GO flakes in liquid medium, albeit most often using low-power bath immersion systems and, therefore, not inducing significant changes in lateral dimensions [11]. The first use of high-power ultrasound treatment for effective reduction of GO’s lateral dimensions was recently reported by Méndez-Romero and coworkers [12]. GO was first produced by the modified Hummers method, having been exfoliated for 1 h in a low-power ultrasonic bath in the last step. A 10 mL amount of this aqueous GO dispersion (with concentration 3 mg mL^−1^) was then submitted to a high-power ultrasound probe (250 W maximum power) for a maximum time of 4 h under controlled temperature (18 °C). The product was centrifuged to separate larger particles, but the authors report that the GOn yield from these ultrasonication and centrifugation steps was 90%. Particle sizes below 100 nm were obtained after 2 h treatment.

The present work further explores the use of high-power ultrasound treatment for efficiently producing GOn. By using a continuous recirculation system, we show that this method can be used for processing large quantities of aqueous GO dispersion at high concentration. In addition, we produce GOn directly from oxidized graphite (GtO), with no intermediate GO exfoliation step in a low-power ultrasonic bath. The final GOn is compared to GO obtained by the conventional centrifugation approach for lateral dimension reduction.

## 2. Materials and Methods

### 2.1. GO Lateral Dimensions Reduction Based on Centrifugation

Graphene oxide (GO) was prepared by oxidation of graphite powder using the modified Hummers method [13]. In a jacketed glass reactor, 160 mL of sulfuric acid (H_2_SO_4_) and 40 mL of phosphoric acid (H_3_PO_4_) were added to 4 g of graphite powder (size ≤ 20 µm, Sigma Aldrich, St. Louis, MO, USA), stirring for 10 min. Then, 24 g of potassium permanganate (KMnO_4_) was slowly added to the solution, which was heated to 35 °C and stirred for 2 h. Following this, 600 mL of H_2_O was gradually added, under stirring. Temperature was controlled using a thermocryostatic bath. To stop the reaction, 26 mL hydrogen peroxide (H_2_O_2_) was added to the mixture. The next day, the solution was decanted and the solid phase was separated from the acidic solution. A centrifugation step was performed at 4000 rpm for 20 min; the solution was redispersed in distilled water, and this protocol was repeated until a neutral supernatant pH was achieved. To obtain well-exfoliated GO flakes, the pellet collected after the last centrifugation step was redispersed in 300 mL of distilled water at a concentration of 1 mg/mL and placed in a conventional ultrasonic bath (Ovan ATM40-3LCD, Barcelona, Spain) with a power density of 30 W/L for 4 h. After sonication, GO dispersion was centrifuged at 13,000 rpm for 30 min, which enabled the separation of two different phases. The supernatant contained the smallest particles and was recovered for later use [3].

### 2.2. GO Lateral Dimensions Reduction Based on High-Power Ultrasonication

**Figure 1:**
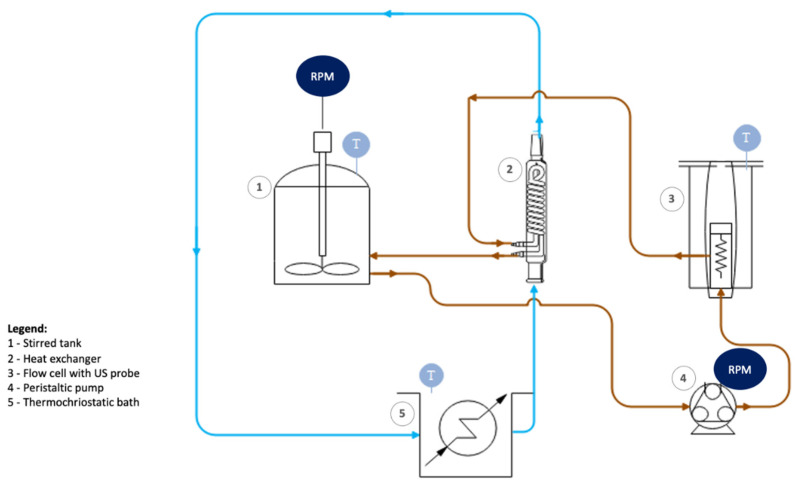
Schematic representation of the recirculation system for GOn production by high-power ultrasonication.

The improved approach relied on a similar oxidation process of graphite powder, as described above, which, after the last washing cycle, was immediately followed by high-power ultrasonication in order to simultaneously exfoliate and breakdown the GO sheets. For this purpose, a high-power ultrasound probe (Hielscher UIP1000hd, 1000 W maximum power, Teltow, Germany) was used (Figure 1). The aqueous dispersion was made to recirculate continuously with the help of a peristaltic pump (Watson-Marlow 323, Falmouth, UK) through a 40 mL stainless steel flow cell (Hielscher FC100L1) housing the ultrasound probe, and a glass condenser that worked as a heat exchanger for cooling. The ultrasound power density in the flow cell was 25,000 W/L, which is coincidentally the same as in the abovementioned work by Méndez-Romero et al., who have also dealt with high-power ultrasonication [12]. Note that in a conventional ultrasound bath, power densities typically range from 10 to 40 W/L. The cooling fluid was water pumped from a thermocryostatic bath kept at 40 °C. The recirculation flow rate was 400 mL/min. Samples were collected at different times to evaluate the influence of process duration on GOn size, up to a maximum of 8 h. The final product was collected and was stored without further processing. A total volume of 1000 mL of GOn dispersion could be processed in this system, with a concentration of 7.5 mg mL^−1^. It was observed that concentrations above 10 mg mL^−1^ were difficult to process due to increased viscosity.

**Figure 2:**
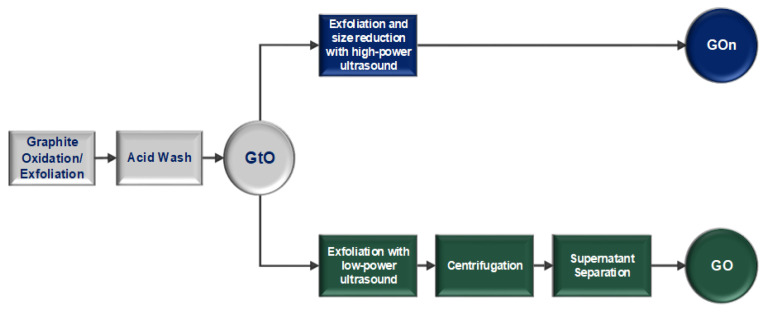
Flow diagram for the new high-power ultrasound method and the conventional centrifugation method for production of GO with small lateral dimensions.

Figure 2 depicts schematically the steps involved in the two production methods used here, which differ in the way how GtO is processed to obtain small size GO. In the ensuing text, we shall designate as “GOn” the graphene oxide produced by high-power ultrasound, and as GO the graphene oxide obtained by the conventional low-power ultrasound plus centrifugation method.

### 2.3. Transmission Electron Microscopy (TEM)

GO and GOn aqueous dispersions were analyzed by transmission electron microscopy (TEM, JEOL JEM 1400 TEM, Tokyo, Japan) to evaluate their morphology and determine lateral dimensions. For this, 10 µL of each sample (concentration of 50 µg mL^−1^) were deposited on Formvar/carbon film-coated 300 mesh nickel grids (Electron Microscopy Sciences, Hatfield, PA, USA) and left standing for 1 min, followed by excess liquid removal with filter paper. Nanomaterial sizes were determined using ImageJ software (version 1.53) by measuring lateral dimensions of platelets using several acquired TEM images [14].

### 2.4. Zeta Potential Measurements

Zeta potentials of GO and GOn aqueous dispersions at a concentration of 25 µg mL^−1^ were measured in a Zetasizer Nano-ZS (Malvern Instruments, Worcestershire, UK) using a disposable Zetasizer cuvette (Malvern Instruments, Worcestershire, UK). All samples were analyzed in triplicate at room temperature. Results are reported as mean and standard deviation.

### 2.5. Fourier Transform Infrared (FTIR) Spectroscopy

GO and GOn dehydrated samples were examined using a VERTEX 70 FTIR spectrometer (Bruker, Karlsruhe, Germany) in transmittance mode. Samples were analyzed in ATR mode using a A225/Q PLATINUM ATR Diamond crystal with single reflection accessory at room temperature (Bruker, Karlsruhe, Germany). Infrared spectra were recorded over the wavenumber range between 4000 and 400 cm^−1^ and 64 scans were averaged at a resolution of 4 cm^−1^.

### 2.6. X-ray Photoelectron Spectroscopy (XPS)

X-ray photoelectron spectroscopy (XPS, Kratos Axis Ultra HSA, Manchester, UK) analysis was performed at CEMUP (Centro de Materiais da Universidade do Porto, Porto, Portugal). Data acquisition was performed using a monochromator Al X-ray source operating at 15 kV (90 W). The survey XPS spectra were acquired, with pass energy (PE) of 80 eV, 1 eV step size, 200 ms dwell time and averaged from two scans. High-resolution C1s and O1s XPS spectra were acquired averaging five scans, with PE of 40 eV, 0.1 eV step size, 1500 ms (for C1s), and 1000 ms (for O1s) dwell time. Spectra were then analyzed using CasaXPS software (Casa Software Ltd., Teignmouth, UK). The contribution of the electric charge was corrected by calibrating all samples to the carbon peak reference at a binding energy of 284.6 eV.

### 2.7. Thermogravimetric Analysis (TGA)

Weight loss of GO and GOn (sample amounts of 4–4.5 mg) was determined by thermogravimetric analysis (TGA) (Netzsh STA 449 F3 Jupiter, Selb, Germany) under a constant temperature increase. All thermograms were obtained under nitrogen flow and collected between 30 and 1000 °C at a heating rate of 10 °C min^−1^. Results are presented as percentage (%) of weight loss.

### 2.8. X-ray Diffraction Analysis (XRD)

GBM films were analyzed by XRD using a Rigaku SmartLab diffractometer (Tokyo, Japan). For data acquisition, an operating voltage of 45 kV and 200 mA under Cu Kα radiation of wavelength *λ*~1.540 Å, in a Bragg–Brentano geometry, was used. GO and GOn films were analyzed in the range of 5–50 theta (*2θ*) with a step of 0.01° and measured in a rotative system (30 deg min^−1^) to increase the crystallite size statistics. Crystallites interlayer d-spacing was extracted from the Rigaku PDXL XRD analysis software based on the Bragg’s law equation.

### 2.9. Raman Spectroscopy

Raman spectroscopy was performed to characterize vibrational GOn modes. Spectra were acquired using a Raman confocal microscope (WITec alpha300 R, Ulm, Germany). Excitation was provided by a 532 nm laser. Measurements were performed using film samples. Each spectrum is an average of 30 scans and was corrected at the baseline and smoothed.

## 3. Results and Discussion

### 3.1. Morphological Features and Dispersion Stability

**Figure 3:**
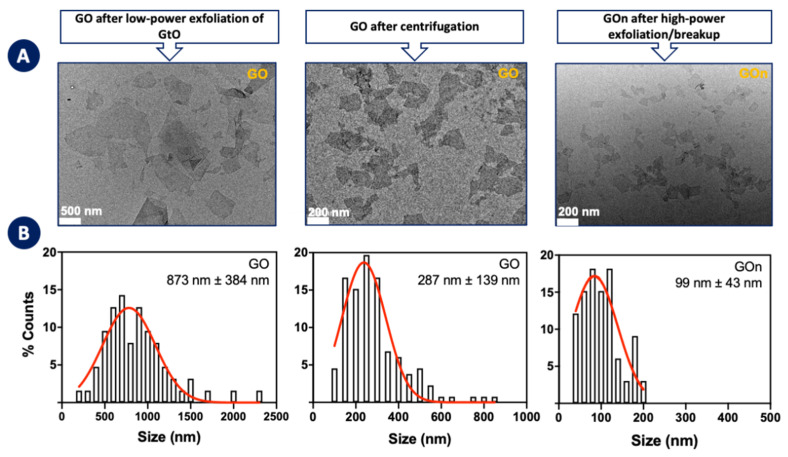
Morphology of GO and GOn. (**A**) Representative TEM images of GO and GOn aqueous dispersions and (**B**) respective particle size distributions and average sizes and standard deviations, as determined from TEM images.

Figure 3 shows TEM images and particle size distributions for GO after low-power ultrasound exfoliation of GtO, GO collected from the supernatant after centrifugation of the previous dispersion, and GOn obtained by direct high-power ultrasonication of GtO.

Centrifugation allows separating GO sheets with 287 nm of average lateral dimensions, lower than the 873 nm of the original GO dispersion, but the global yield, from GtO to the final GO, is only 17%. On the other hand, high-power ultrasonication of GtO allows obtaining GO sheets with lower lateral sizes (average length 99 nm), and a much higher global yield of 74%, since all the treated material is usable, except for process losses (material not properly removed from tubing connections, for instance).

**Figure 4:**
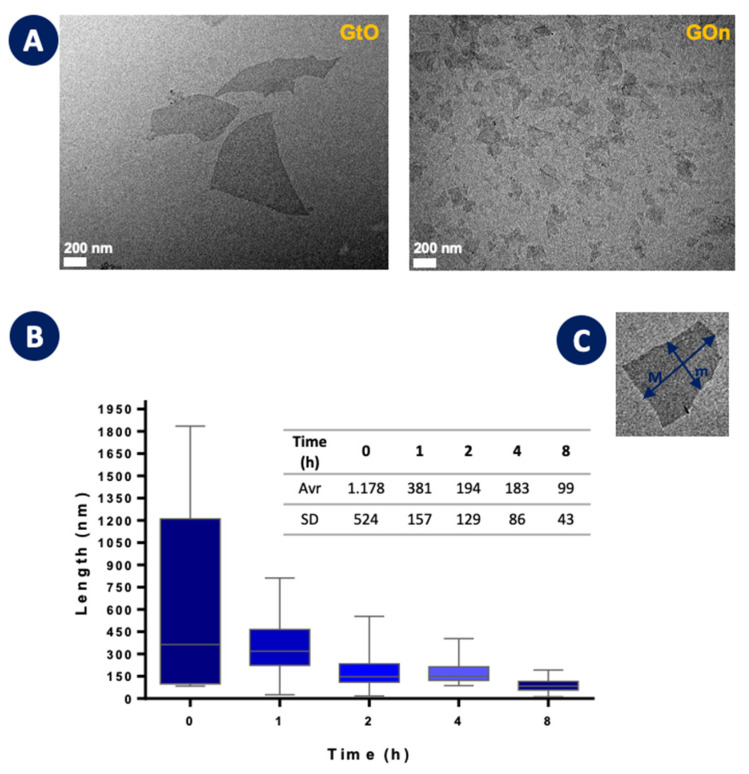
Effect of high-power ultrasonication time on the particle size of GOn. (**A**) TEM images comparing starting GtO particles and the GOn sheets obtained after 8 h of high-power ultrasonication. (**B**) Box plot of particle sizes and average and standard deviations (SD). (**C**) Representation of the major (M) and minor (m) axis used for calculation of average lateral dimensions from TEM images.

Figure 4 shows the evolution of particle size along the high-power ultrasonication step. As noted before, the starting material (time 0 h) is the unexfoliated oxidized graphite. Figure 4A presents TEM images of the starting particles and of the product obtained after 8 h ultrasonication, illustrating the magnitude of lateral size reduction. This process is able to break down particle sizes rapidly and uniformly, eliminating the need for separation of the smaller sizes from the rest of the material. It is noticeable that most of the lateral dimension reduction occurs in the first 2 h (Figure 4B). This fast initial effect was also reported in the abovementioned work by Méndez-Romero and coworkers [12]. The initially large concentration of defects in graphene oxide’s basal plane (lower dissociation energy related to sp^3^ bonding) facilitates sheet breakup caused by the high shear forces associated with ultrasound’s acoustic cavitation.

**Figure 5:**
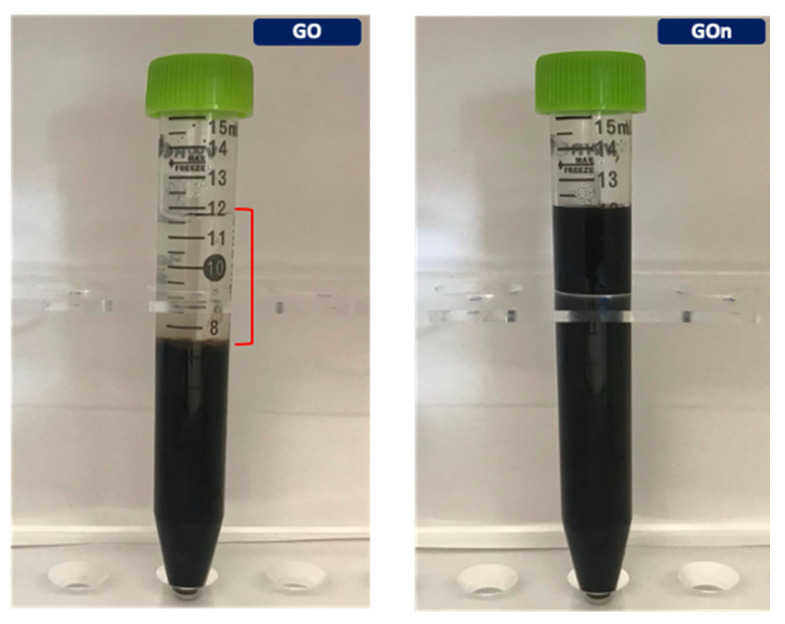
GO (**left**) and GOn (**right**) dispersions after 6 months storage at rest. The red bracket on the left image shows the height of clear supernatant liquid formed in the GO dispersion.

Decreasing GO’s lateral dimensions is expected to lead to higher charge density, due to higher edge-to-area ratio (cationic carboxylic groups are more abundant along the sheet edges). As a consequence, zeta potential as well as colloidal stability tends to be higher [15]. Zeta potential measurements demonstrated that both GO and GOn were negatively charged colloidal particles. As seen in Table 1, we found that high-power ultrasonicated GOn displayed a greater negative surface charge (−39.9 ± 2.2 mV) as compared to centrifuged GO (−29.7 ± 1.2 mV). This higher density of negative electrostatic charges resulted in increased colloidal stability of GOn aqueous dispersions (Figure 5), which exhibited long term stability, even after 6 months storage. On the other hand, GO dispersions were already extensively separated after 6 months (Figure 5), having started to visibly sediment after only one week. Even more striking is the fact that the dispersion concentration was significantly higher for GOn than for GO (7.5 and 1 mg mL^−1^, respectively). Méndez-Romero and coworkers reported that zeta potential did not tend to change with lateral size for their GOn produced by high-power ultrasonication, though they did not evaluate colloidal stability [12].

Generally, smaller average particle size and coefficient of variation (ratio of the standard deviation to the mean particle size) together with lower zeta potential values have been identified as critical parameters to demonstrate dispersion stability of GO materials, thus favoring the production of homogeneous GO dispersions at larger scales and with improved product quality [16,17]. Overall, high-power ultrasonication enabled the production of GO nanosheets with smaller average particle size (99 versus 287 nm for centrifuged GO) and slightly lower smaller coefficient of variation (43% versus 48%). Additionally, GO dispersions exhibiting a zeta potential value lower than −30 mV have been described as highly stable [18,19], further supporting dispersion stability improvement reported herein.

### 3.2. Chemical Properties

**Figure 6:**
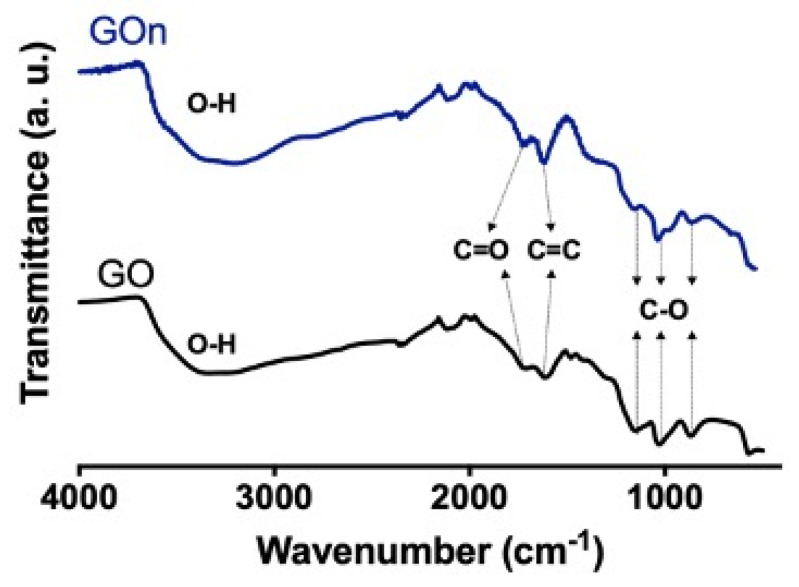
FTIR spectra of GO (black line) and GOn (blue line), showing the contribution of surface functionalities.

Fourier transform infrared (FTIR) spectroscopy was used to analyze the contribution of oxygen functionalities on the surface of GO and GOn (Figure 6). Herein, a broad band could be identified for both GO types in the wavenumber range of 3000 and 3600 cm^−1^, corresponding to O−H stretching vibrations, which are characteristic of adsorbed water molecules and hydroxyl and carboxyl groups [3,14]. A peak was evident at around 1725 cm^−1^ in both GO and GOn spectra, which is assigned to C=O stretching vibrations, demonstrating the presence of carbonyl and carboxyl groups [3,14,20]. The stretching of cyclic alkene (C=C) was observed at approximately 1610 cm^−1^, which results from the unoxidized graphitic backbone [20,21,22]. C−O stretching vibrations attributed to ethers were present at around 1140 and 1020 cm^−1^, and C−O bending vibrations of epoxides were found at around 858 cm^−1^ [3,14]. Further characterization regarding carbon and oxygen contents and chemical functional groups quantification were performed by other techniques, which is presented next.

**Figure 7:**
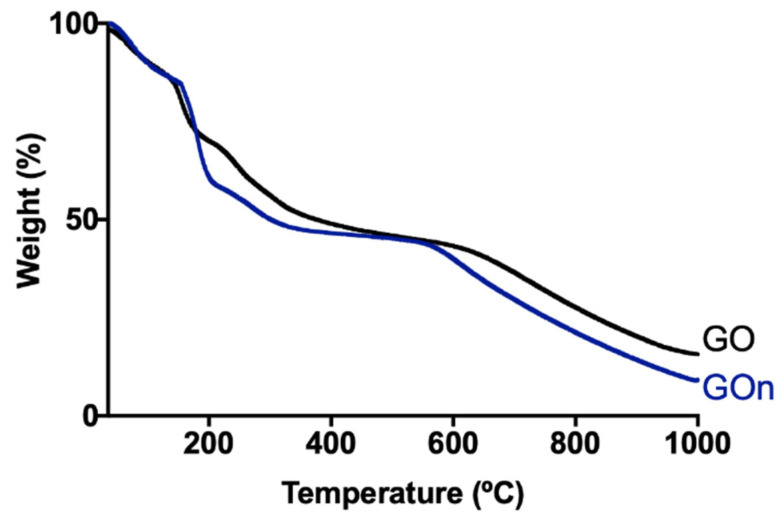
Thermal decomposition of GOn and GO. TGA curves and weight loss values for GOn (blue line) and GO (black line).

GO and GOn functionalization degree and thermal stability were assessed by TGA. Results are shown in Figure 7, displaying the weight loss during heating. Thermograms revealed two main weight loss steps for both materials. The first weight loss occurred between 142–240 °C, which is attributed to the loss of reactive oxygen-containing functional groups, namely carboxyl and epoxy [14,23]. The second weight loss step occurred between 225–600 °C, corresponding to the combustion of carbon skeleton and pyrolysis of more stable functionalities like carbonyls and residual hydroxyls [24]. At 240 °C, GO presented a wt.% loss of 35.1%, while GOn presented a wt.% loss of 43.6%. At 600 °C, GO presented a wt.% loss of 56.8%, while GOn presented a wt.% loss of 60.0%. Such results demonstrate that GOn might be slightly more reduced than GO. Our method involves recirculation of GtO dispersions through a high-power sonication probe for 8h. Even though the fluid is cooled during the entire process, as the material contacts with the tip several times, slight chemical reduction might be occurring. In addition, in GO production, there is a high-speed sonication step (13,000 rpm) that allows collecting the material in the supernatant composed of the more water-stable particles, therefore with more oxygen-containing functional groups.

**Figure 8:**
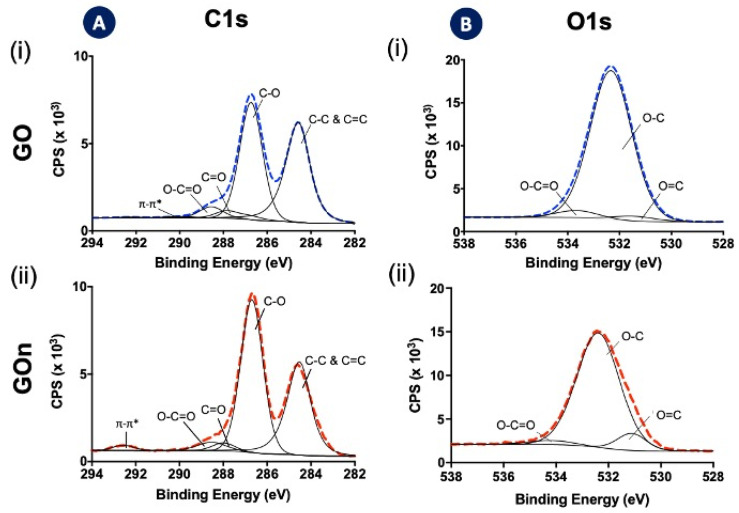
XPS analysis of (i) GO and (ii) GOn. Deconvolution of high-resolution (**A**) C1s and (**B**) O1s spectra enabled the quantification of functional groups on graphene backbone.

X-ray photoelectron spectroscopy (XPS) analyses allowed the quantification of the oxidation degree and surface functionalization of GO and GOn (Figure 8, Table 2). GO exhibited a C at. % of 62.1 and a O at. % of 32.0, whereas GOn presented a slightly higher C at. % of 66.3, together with a O at. % of 30.7 (Table 2). These results indicate successful oxidation and introduction of oxygen functionalities at the surface of GO and GOn. Moreover, similar C/O ratios were determined for both GO types (1.94 and 2.16 for GO and GOn, respectively), suggesting a slightly higher oxidation degree for GO than GOn (Table 2). As previously mentioned, the higher energy applied during contact with the high-power sonication probe in multiple recirculation cycles could have contributed to a slight reduction in GOn. Similarly, Méndez-Romero and coworkers reported a slight increase in C/O ratio, ranging from 1.95 to 2.01, for their GOn exposed to increasing times of high-power ultrasonication [12]. It is important to notice that, in our method, we produce GOn directly from GtO, contrary to the previously mentioned work, where previously exfoliated GO (not GtO) is size reduced.

Analysis of C1s spectra of both GO and GOn showed two large peaks, which were then deconvoluted in five peaks centered at 284.5, 286.7, 287.9, 288.5, and 292.6 eV (Figure 8A, Table 2), which can be attributed to formation of the following:

1. sp^2^ and sp^3^ hybridizations of carbon (C–C and C=C, C1s at. % = 45.5 for GO and C1s at. % = 38.4 for GOn) in the graphitic backbone;
2. single bonds between carbon and oxygen (C–O) in hydroxyls and ethers (C1s at. % = 44.9 for GO and C1s at. % = 53.2 for GOn);
3. double bonds between carbon and oxygen (C=O), indicating the presence of carbonyl groups (C1s at. % = 4.2% for GO and C1s at. % = 2.8% for GOn);
4. multiple bonds between carbon and oxygen (O=C−O), indicating the occurrence of carboxyls (C1s at. % = 4.5 for GO and C1s at. % = 3.9 for GOn); and
5. π–π^*^ bonds due to the presence of delocalized π electrons in the graphene lattice (C1s at. % = 0.93 for GO and C1s at. % = 1.8 for GOn) [3,14,25].

The high-resolution O1s spectra were deconvoluted in three peaks centered at 531.2, 532.5, and 534 eV (Figure 8B, Table 2), which can be also attributed to formation of the following:

1. O=C bonds present in carbonyl and carboxyl groups (O1s at. % = 3.1 for GO and O1s at. % = 5.7 for GOn);
2. O–C bonds in hydroxyl groups and ethers (O1s at. % = 92.4 for GO and O1s at. % = 92.5 for GOn); and
3. O–C bonds from carboxyls (O=C–O, O1s at. % = 1.9 for GO and O1s at. % = 4.5 for GOn) [3,14,26].

The relative percentage of chemical bonds found in both C1s and O1s spectra are in accordance. The analysis of the deconvoluted spectra showed that GO (Figure 8A) and GOn (Figure 8B) were well oxidized, as demonstrated by the existence of carbon atoms in functional groups (hydroxyl, carbonyl, and carboxyl) with C−O bonds dominating the surface chemistry. Despite a slightly lower abundance of C–O bonds being found in the C1s spectra of GOn (Figure 8A), which resulted in a higher abundance of C–C bonds compared to GO, the analysis of the O1s spectra (Figure 8B) showed similar amounts of oxygen atoms involved in C–O bonds between both GO materials. Altogether, these results demonstrate that both GO and GOn exhibited high degree of oxidation, in accordance to other studies about the quality of GO materials reporting C/O ratios in the range between 1.94 and 2.6 [12,14,23].

**Figure 9:**
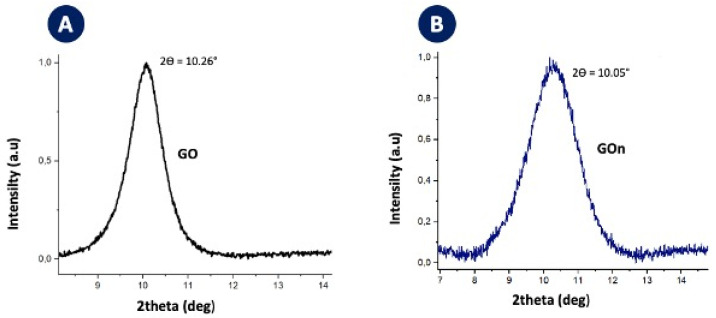
XRD patterns of (**A**) GO and (**B**) GOn, corresponding to characteristic network reflections.

The materials have also been characterized by XRD, showing typical spectra with GO presenting a 2*θ* angle of 10.26° (Figure 9A) and GOn presenting a value of 10.05° (Figure 9B). Interlayer spacing for GO was of 8.8 Å, while GOn presented an interlayer spacing of 8.6 Å. This confirms full graphite oxidation and successful exfoliation into GO or GOn [27].

**Figure 10:**
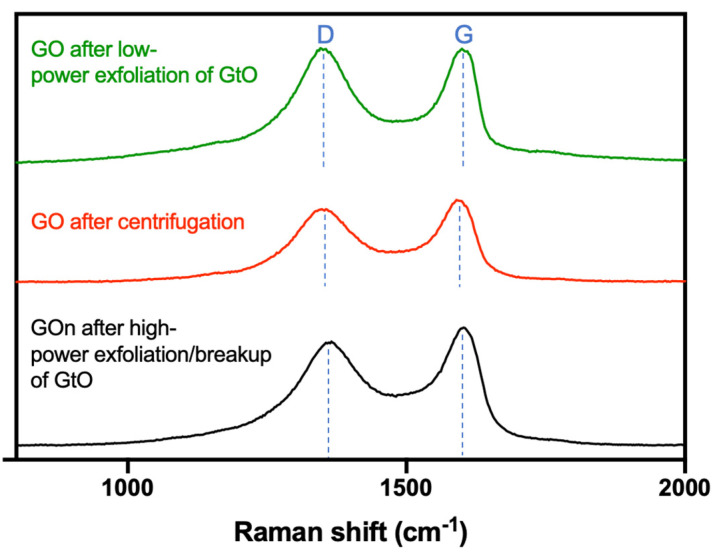
Raman spectra of GO after low-power exfoliation, GO after centrifugation, and GOn after high-power exfoliation/breakup of GtO.

Raman spectroscopy was used to further characterize the produced GO types. Graphene-based materials typically present spectra with marked D and G bands [28]. The D peak results from the presence of vacancies or dislocations in the graphene basal plane and at its edge, being therefore related to the presence of structural defects [28,29]. Raman spectra obtained demonstrated the appearance of defects in the crystal structure of graphene, as seen by the D band (Figure 10), which was observed at 1355 cm^−1^ for GO after low-power exfoliation of GtO, 1358 cm^−1^ for GO after centrifugation, and 1365 cm^−1^ for GO after high-power exfoliation of GtO (Table 3). The disorder D band is a typical feature of graphene oxide as a result of oxidation [14,23,28]. The G band, which is related to the in-plane vibration of sp^2^ hybridized carbon atoms [28,29], was also present in all samples (Figure 10), being identified at 1605 cm^−1^ for GO after low-power exfoliation of GtO, 1600 cm^−1^ for GO after centrifugation, and 1602 cm^−1^ for GO after high-power exfoliation of GtO (Table 3).

Ratios between the bands can provide further information on materials structure. The *I*_D_/*I*_G_ ratio is related to the amount of defects present in the graphitic structure [29]. The *I*_D_/*I*_G_ ratio was highest for GO after low-power exfoliation of GtO, but decreased as a result of high-power ultrasonication (0.998 versus 0.880, Table 3). High-power sonication has been reported to increase the number of grain boundaries, as particle size is reduced, therefore an increase in *I*_D_/*I*_G_ ratio would be expected. However, we have observed that for our particular method, the C/O for GOn is slightly smaller than for GO. This might have resulted in the presence of less defects in GOn basal plane carbon structure, derived from oxygen-containing functional groups presence, contributing to *I*_D_/*I*_G_ ratio reduction [12].

## 4. Conclusions

In this work, we reported the use of high-power ultrasonication for one-step graphite oxide exfoliation and breakup in order to produce graphene oxide sheets with very small lateral dimensions. We characterized the impact of this process on the physicochemical properties of the product, comparing it to the material obtained via centrifugation of conventionally produced GO (modified Hummers method followed by low-power ultrasonic exfoliation). The continuous recirculation setup used allowed for processing large amounts of highly concentrated GtO dispersion (up to 1 L at 7.5 mg/mL) at a controlled temperature, producing GOn with average lateral dimensions of 99 nm with a process yield of 74%. This particle size reduction method resulted in more stable GO nanocolloids, withstanding a 6-month period of shelf-life owing to the higher charge density, which was demonstrated by a more negative zeta potential of GOn as opposed to the GO sheets separated by centrifugation. Oxygen-containing functional groups were identified in similar quantities for both GO types, indicating that the size breakup treatment did not affect the degree of oxidation. This shows that high-power ultrasound treatment is an effective, productive, and expedited method for manufacture of nanocolloidal GO that can easily be scaled up for industrial production.

## Figures and Tables

**Table 1:** Zeta potential measurements of GO and GOn aqueous dispersions.

GBM	Surface Charge (mV)
GO	−29.7 ± 1.2
GOn	−39.9 ± 2.2

**Table 2:** Atomic composition of GO and GOn and content of C1s and O1s chemical functional groups resulting from XPS spectra fitting.

Elemental at. %/Chemical Group		Binding Energy (eV)	GO (%)	GOn (%)
	C/O ratio	-	1.94	2.16
	C1s	-	62.1	66.3
	O1s	-	32.0	30.7
C 1s (at. %)	C–C and C=C	284.5	45.5	38.4
	C–O	286.7	44.9	53.2
	C=O	287.9	4.2	2.8
	O=C–O	288.5	4.5	3.9
	π–π*	292.6	0.93	1.8
O 1s (at. %)	C=O	531.2	3.1	5.7
	C–O	532.5	92.4	92.5
	O=C–O	534	4.5	1.9

**Table 3:** Position of the band (cm^−1^) from Raman to GO after low-power exfoliation, GO after centrifugation and GOn after high-power exfoliation/breakup pf GtO and respective I_D_/I_G_ band ratios.

Samples	D Band	G Band	*I*_D_/*I*_G_ Band
GO after low-power exfoliation of GtO	1355	1605	0.998
GO after centrifugation	1358	1600	0.968
GO after high-power exfoliation/breakup of GtO	1365	1602	0.880

## Data Availability

The data presented in this study are available on request from the corresponding author.
